# Green synthesized gold nanoparticles from *Pseudobulbus Cremastrae seu Pleiones* show efficacy against hepatic carcinoma potentially through immunoregulation

**DOI:** 10.1080/10717544.2022.2092238

**Published:** 2022-06-28

**Authors:** Junmo Zhu, Zijing Liu, Youwei Pu, Jie Xu, Sitong Zhang, Yixi Bao

**Affiliations:** a Department of Clinical Laboratory, The Second Affiliated Hospital of Chongqing Medical University, Chongqing, China; b Department of Gastroenterology, The Third Affiliated Hospital of Chongqing Medical University, Chongqing, China

**Keywords:** *Pseudobulbus Cremastrae seu Pleiones*, gold nanoparticles, green synthesis, immune function, liver cancer

## Abstract

Nanobiotechnology, the interface between biology and nanotechnology, has recently emerged in full bloom in the medical field due to its minimal side-effects and high efficiency. To broaden the application of nanobiotechnology, we composed gold nanoparticles from the extract of *Pseudobulbus Cremastrae seu Pleiones* (PCSP) using an efficient and green procedure. The biosynthesized Au nanoparticles containing PCSP (PCSP-AuNPs) were characterized by UV-vis spectroscopic, transmission electron microscopy (TEM), atomic force microscopy (AFM), dynamic light scattering (DLS), Fourier transform infrared spectroscopy (FT-IR), and Energy Dispersive X-ray (EDAX). After verifying the stability of PCSP-AuNPs, we detected its biosafety and immune-modulatory effects on RAW264.7 *in vitro* using NO assay, ELISA (TNF-α, IL-12p70, and IL-1β), and CCK-8 test. Furthermore, we examined the direct *in vitro* effects of PCSP-AuNPs on hepatocellular carcinomas (HCCs). Finally, we evaluated the immune regulation of PCSP-AuNPs using a mouse model with H22-tumor by testing the index of immune organs, splenic lymphocyte proliferation, cytokines levels (TNF-α and IL-10), and the CD4+/CD8+ cell ratio in the peripheral blood. Immunohistochemical analyses including H&E and PCNA staining were performed to investigate the anti-cancer efficacy and biocompatibility of PCSP-AuNPs. We found that PCSP-AuNPs not just possessed low toxicity, but also improved the immune-mediated antitumor response as compared to PCSP alone, suggesting its potential as a novel and efficient drug for liver cancer therapy.

## 1. Introduction

Nanomedicine, a rapidly developing discipline, has found application in the treatment of various conditions such as inflammation, cancer, and immune system disorders. Instead of the physicochemical methods for synthesizing nanoparticles, currently, there is a push toward environment-friendly and biogenic nanomaterials, thereby, referring to the biodegradable characteristics of plants for generating green nanoparticles (Wang et al., 2021). In recent years, among all noble metal nanoparticles, gold nanoparticles have received extensive attention owing to their biocompatibility, tunable surface plasmon resonance (SPR) and unique optoelectronic characteristics (Boisselier & Astruc, 2009; Zeiri et al., 2014). It has been reported that gold nanoparticles together with the micro-environmental stimuli could help activate the immune system (MacParland et al., 2017; Al-Omar et al., 2021). Furthermore, gold nanoparticles possess a rough surface with a high surface-to-volume ratio, enabling these particles with high cohesive ability to form various biomolecules, providing us an opportunity to biosynthesize nanocomposites with less toxicity (Ahn et al., 2018; Khan et al., 2020).

Over the past decade, Traditional Chinese medicines (TCM) have played a key role in cancer immunotherapy. Li et al. (2020) reported that Astragalus polysaccharide inhibited breast cancer growth by regulating the immune response. The capacity of shikonin for activating anti-tumor immunity was investigated by Lin et al. (2015) Many of the previously published studies have paid attention to herbal medicines due to their low toxicity and high efficacy. *Pseudobulbus Cremastrae seu Pleiones* (PCSP), a pseudobulb of orchid plants is a typical TCM that has been used for clearing heat and detoxification, and for eliminating carbuncle and dispelling knots, etc. It has been reported that PCSP could stimulate the immune response and exhibited anticancer effects (Fang et al., 2018). Nevertheless, when administered alone, it suffered from several limitations such as the nonspecific scope of action, high dosage, and low bioavailability. Therefore, we combined TCM with emerging nanotechnology to fabricate the novel biosynthesized nanocomposite PCSP-AuNPs to evaluate whether it could improve anti-tumor efficacy.

There are many reports about TCM such as Ganoderma lucidum (Zhang et al., 2019), Astragalus membranaceus (Pang et al., 2019), and Curcuma wenyujin (Liu et al., 2019), bound to gold nanoparticles to treat various types of cancer. The liver is the sixth most common site of primary cancer in humans and usually appears in the context of cirrhosis and inflammation. Moreover, the liver is often colonized by cancer metastases from other organs, especially the colon (Li et al., 2021). Patients are often diagnosed with liver cancer at an advanced stage, resulting in a poor prognosis. Hepatocellular carcinomas (HCCs) account for 90% of all liver cancer cases, wherein surgical resection and chemotherapy are generally the treatment of choice. However, they not only have limited efficacy in improving the prognosis of liver cancer, but also cause great harm to the patient’s body (Forner et al., 2018; Anwanwan et al., 2020). Consequently, novel therapeutic options for treating liver cancer are urgently needed.

**Figure 1.**
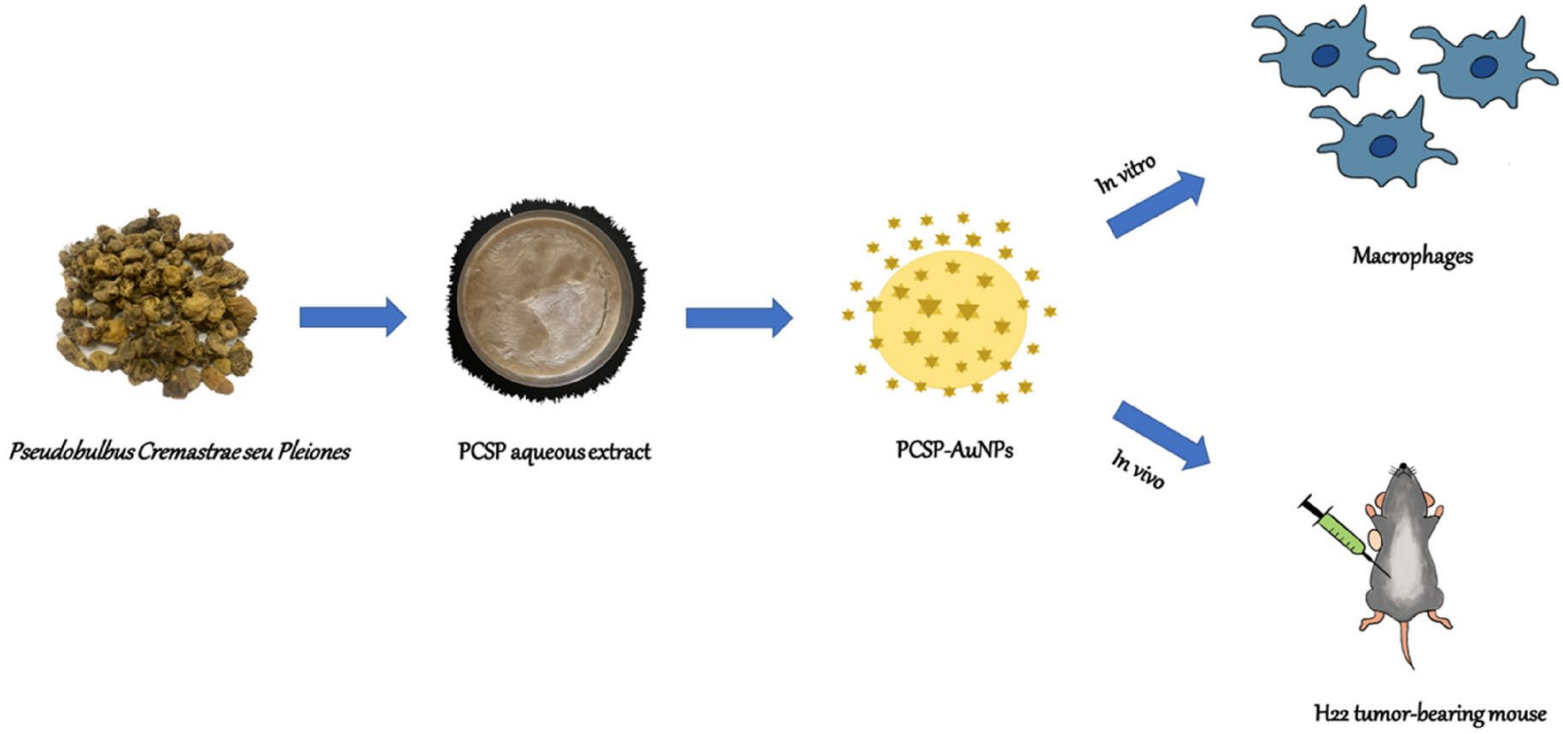
Schematic diagram of the study content of PCSP-AuNPs.

As mentioned above, the application of natural Chinese medicine and nanobiotechnology may enable patients to have a better prognosis, fewer side effects, and lower systemic toxicity. Therefore, in this study, we employed PCSP and HAuCl4 to biosynthesize a novel gold nanocomposite, PCSP-AuNPs, and evaluated its immune-enhancing and anti-tumor effects, which may serve as a novel potential therapeutic agent against liver cancer (Figure 1).

## 2. Materials and methods

### 2.1. Reagents

*Pseudobulbus Cremastrae seu Pleiones* were procured from a local Chinese pharmacy. Beijing G-CLONE Biological Technology Co.Ltd. (Beijing, China) provided trisodium citrate dihydrate. Chloroauric acid (HAuCl_4_) was obtained from Shanghai Xianding Biological Technology Co.Ltd. (Shanghai, China). EDTA/trypsin and Dulbecco’s modified Eagles medium (DMEM) were products of Hyclone (Logan City, UT, USA). Fetal bovine serum (FBS) was sourced from Gibco Life Technologies (Grand Island, USA). Adriamycin (ADM) was bought from Shenzhen Main Luck Pharmaceuticals Inc. (Shenzhen, China), whereas Lipopolysaccharide (LPS) was from Sigma–Aldrich (St. Louis, MO, USA), NO assay kit was purchased from Beyotime Biotechnology (Shanghai, China), and ELISA kits for mouse TNF-α, IL-12p70 and IL-1β were obtained from 4 A Biotech (Beijing, China). Boster Biological Technology Co.Ltd. (CA, US) supplied the Cell counting kit-8 (CCK-8) test kit and RPMI1640 media, while BD Pharmingen (CA, USA) supplied the anti-CD3, anti-CD4, and anti-CD8 antibodies.

### 2.2. Preparation of PCSP-AuNPs

Firstly, we prepared *Pseudobulbus Cremastrae seu Pleiones* aqueous extract by boiling, filtering, concentrating and freeze-drying, to obtain PSCP powder for further research. The PSCP powder was dissolved in distilled water and stirred at room temperature for 10 minutes for uniform dispersion. We continuously adjusted the PCSP concentration (from 10 mg/mL to 1 mg/mL), volume (from 2 mL to 0.4 mL), amount of HAuCl_4_ (3 μL, 2.5 μL, 2 μL, 1.5 μL, 1.25 μL, 1 μL) and the reaction time (10 min, 15 min, 30 min, 45 min). We adopted the control variable method, determining one parameter at a time, and then changing the other parameters to optimize the conditions for the green synthesis of PCSP-AuNPs. The formation of PCSP-AuNPs was preliminarily evaluated by the color of the solution changing from light yellow to ruby red (Liu et al., 2019), following which we carried out further confirmation tests. Besides, we synthesized gold nanoparticles with trisodium citrate dihydrate (AuNPs) based on the previously published Turkevich method (Turkevich et al., 1951; Frens, 1973), to rule out the experimental effects of AuNPs without PCSP.

### 2.3. Characterization of PCSP-AuNPs

PCSP, PCSP-AuNPs and AuNPs were subjected to Ultraviolet-Visible (UV-vis) spectroscopic analysis in the range of 200-800 nm to further detect the synthesis of PSCP-AuNPs. We performed this operation for PSCP-AuNPs over a period of 24 hours to 28 days and observed changes in the absorption peaks to measure the stability of the compound. Transmission Electron Microscopy (TEM) and atomic force microscope (AFM) were used to examine the morphological characteristics. In addition, Dynamic light scattering (DLS) measurements, Fourier-transform infrared (FTIR) spectroscopy, and the Energy Dispersive X-ray (EDX) analysis were applied to further characterize PCSP-AuNPs.

### 2.4. Cells culture

The RAW264.7 macrophages, HCC cell lines (HepG2, Huh7, and H22) and L02 cells were obtained from the Department of Hepatobiliary Surgery at the Second Affiliated Hospital of Chongqing Medical University. L02 cells were routinely cultured in RPMI-1640 medium, while the other cells were cultured in DMEM. Both media were supplemented with 10% FBS, 100 U/mL of penicillin and 100 μg/mL of streptomycin at 37 °C in a 5% CO2 atmosphere.

### 2.5. Measurement of the release of NO and cytokines from murine macrophages

The levels of nitric oxide (NO) and cytokines in the macrophage supernatant were determined using the Griess and ELISA methods, respectively. In brief, RAW264.7 macrophages were plated onto a 24-well plate (5 × 10^4^ cells/well) for at least 4 hours to allow the cells to adhere to the plate. Then, LPS (100 ng/mL) and varying concentrations (10, 20, 30, 40, 50 μg/mL) of PCSP, PCSP-AuNPs and AuNPs were applied to each well and incubated for 24 hours. Following that, supernatants were collected after centrifuging the culture medium at 2000 rpm for 20 minutes, and were analyzed with the Griess kit or the ELISA kit according to the manufacturer’s instructions. The NO level was calculated from the absorbance at 540 nm measured by a microplate reader, while the levels of the cytokines, TNF-α, IL-12p70, and IL-1β, were determined by measuring the absorbance at 450 nm.

### 2.6. Direct effect of PCSP-AuNPs

We conducted the CCK-8 test to analyze the effect of PCSP-AuNPs on the viability of RAW264.7 and L02 cells, as well as its direct effect on HCC cell lines (HepG2 and Huh7 cells). RAW264.7, L02, HepG2, and Huh7 cells were separately seeded in 96-well plates at a density of 5 × 10^3^ cells/well. After adhesion, a range of concentrations (10-50 μg/mL) of PCSP, PCSP-AuNPs, and AuNPs acted on the cells for 24 hours. The cell viability was obtained by the CCK-8 kit. The following formulae were used for calculations:

Cell viability (%) = ([OD (experiment)−OD (Blank)])/([OD (control)−OD (blank)]) × 100%

Cell inhibition rate (%) = ([OD (control)−OD (experiment)])/([OD (control)−OD (blank)]) × 100%

### 2.7. Animal modeling and antitumor treatments

We purchased female C57BL/6 mice (weight: 18–22 g, age: 4–6 weeks) from the Animal Facility at the Chongqing Medical University. After a week-long acclimatization, a total of 4 × 10^5^ H22 cells were inoculated subcutaneously into the right armpit of each mouse. The mice were randomly divided into four groups with five mice in each group after successful establishment of the subcutaneous tumors. Intraperitoneal injection was adapted for each treatment and the groups were as follows: (1) normal saline (NS) group (daily, 200 μL/mouse); (2) PCSP group (daily, 20 mg/kg); (3) PCSP-AuNPs group (daily, 20 mg/kg); and (4) ADM group (once every three days, 4 mg/kg). Body weight and tumor size were measured every 2 days with a caliper and the tumor volume was calculated using the following formula: ([major axis] × [minor axis] (Boisselier & Astruc, 2009))/2. Meanwhile, during the experiment, we continuously observed the activity and mental state of all the mice.

After 20 days, the mice were sacrificed. Peripheral blood was collected and divided into two parts. Anticoagulant was added to one part, according to the instructions, followed by mixing with anti-CD3/CD4/CD8 antibodies, incubation in the dark, removal of erythrocytes, washing with PBS, and then detection by flow cytometry (Beckman coulter, Navios, USA). The other part was used to collect serum for cytokines analysis (TNF-α and IL-10), as well as for conducting liver and kidney function tests.

At the same time, the tumors and some organs were collected for different analyses. We weighed the tumors, spleen, and thymus to calculate the tumor inhibition rate and the organ indexes as follows:

Tumor inhibition rate = [1 − a/b] 100%,

a: the weight of the tumors of the treated groups. b: the average weight of the tumors of the NS group.

Organ index = spleen (thymus) weight/body weight × 100% (100‰)

All the tumors were preserved using 4% polyoxymethylene and were subjected to hematoxylin & eosin (H&E) and proliferation cell nuclear antigen (PCNA) staining. We randomly found three fields at × 400 magnification from the images using the OLYMPUS BX53 microscope to calculate the proliferation index of the tumors. Meanwhile, the heart, liver, spleen, lung, and kidney were stored in the same way as the tumors and were stained with H&E to assess the systemic toxicity of the treatments, together with biochemical assays.

In addition, the mouse spleen was collected aseptically, and the splenic lymphocytes were extracted following the standard operation. The splenocytes were suspended in a 96-well plate (5 × 10^5^ cells/mL) with different concentrations (10-50 μg/mL) of PCSP and PCSP-AuNPs for 48 h. The CCK-8 solution was mixed to each well for 4 h and the viability of lymphocytes was evaluated by measuring the optical density at 450 nm (OD 450 nm).

### 2.8. Statistical analysis

The results were analyzed by one-way ANOVA using IBM SPSS Statistics 26.0. All the data given were displayed as the mean ± standard deviation (SD), evaluated from three independent experiments. *p* < 0.05 or *p* < 0.01 indicated statistical significance.

## 3. Results and discussion

### 3.1. Synthesis and characterization of PCSP-AuNPs

#### 3.1.1. Uv-visible spectroscopy analysis

**Figure 2.**
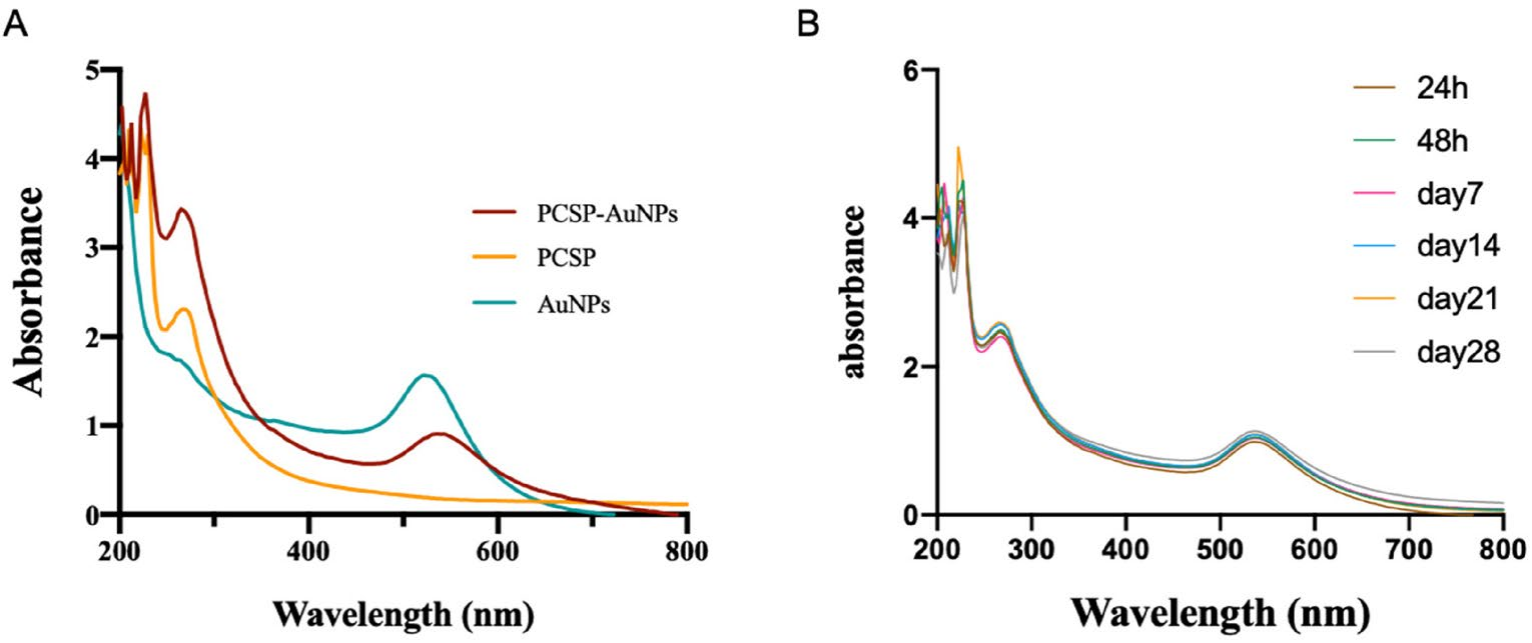
The UV-vis absorption spectra (200-800 nm). (A) The UV-vis spectra for PCSP, PCSP-AuNPs, and AuNPs. (B) The UV-vis spectra of PCSP-AuNPs at different time periods.

Variables affecting the formation of gold nanoparticles including temperature, reaction time, and concentrations of PCSP and HAuCl_4_, were constantly adjusted. We used UV-vis spectra to confirm the successful synthesis of PCSP-AuNPs after the color variation. The classical surface plasmon resonance (SPR) band is around 540 nm, suggesting the formation of AuNPs (Pang et al., 2019). As illustrated in Figure 2A, there were two absorption peaks, one peak was at ∼540 nm for AuNPs and the other peak at ∼260 nm was indicative of PCSP, confirming the successful green synthesis of the nanocomposite only from PCSP and gold. Moreover, the wave crests were basically constant from day 1 to day 28, and no apparent difference was presented in the SPR with an increase in time (Figure 2B). Finally, we optimized the conditions to synthesize stable PCSP-AuNPs: 1.2 mL, 2 mg/mL of PCSP reacted with 2 μL, 1 M of HAuCl_4_ at 80 °C for 15 minutes.

#### 3.1.2. Size and morphology of PCSP-AuNPs

**Figure 3.**
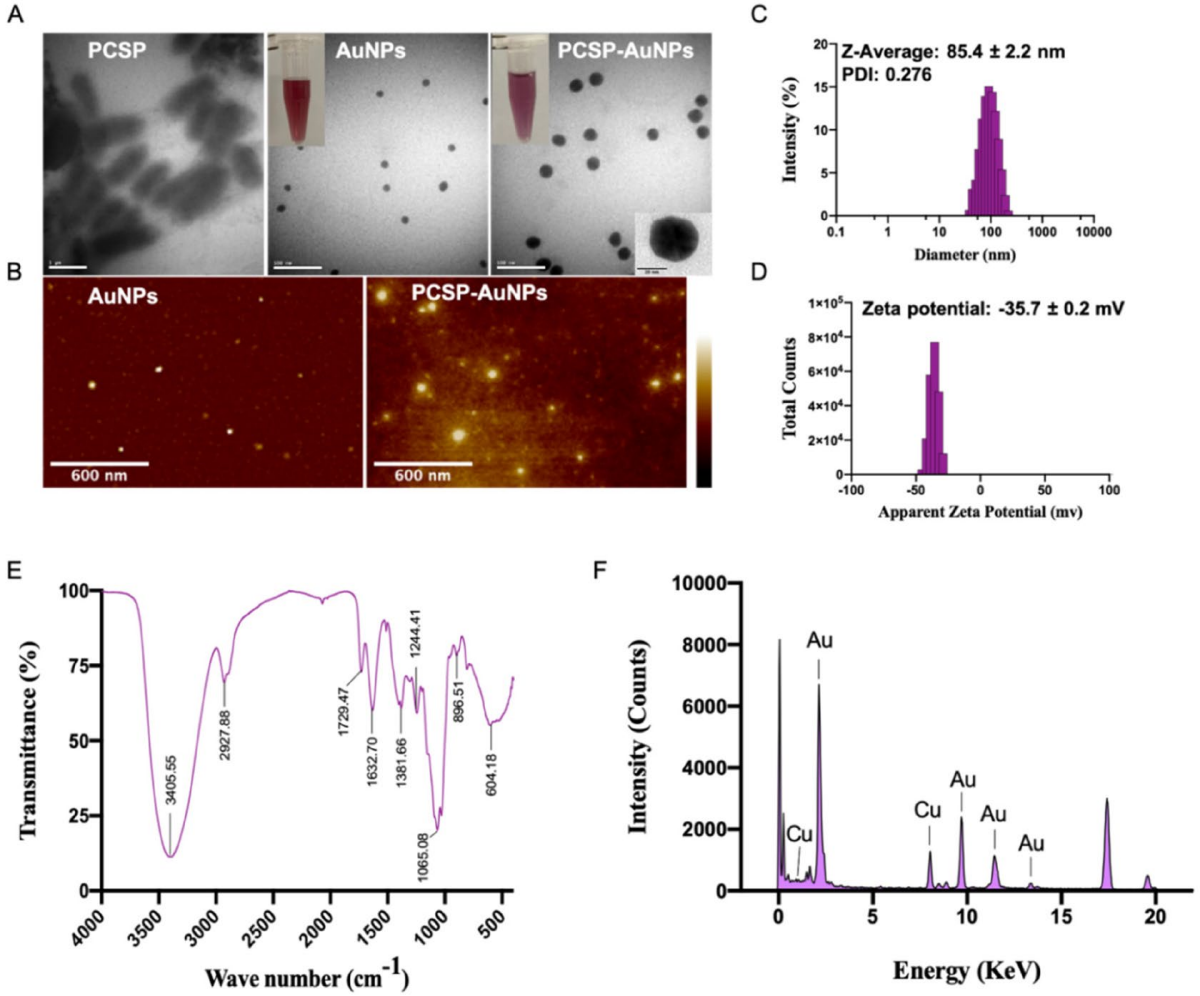
Characterization of PCSP-AuNPs. (A) TEM images of PCSP, AuNPs, and PCSP-AuNPs. (B) AFM analysis of AuNPs and PCSP-AuNPs. (C, D) Dynamic light scattering (DLS) analysis. (E) Fourier-transform infrared (FTIR) spectroscopic analysis. (F) Energy Dispersive X-ray analysis (EDAX).

The TEM graph (Figure 3A) of PCSP-AuNPs confirmed the presence of uniform spherical particles with the diameter of 30.4 ± 3.36 nm, which were larger in comparison to the AuNPs without PCSP. We observed a noticeable difference in the TEM image of PCSP before the nanoparticles were synthesized. The AFM image of the biosynthesized PCSP-AuNPs is depicted in Figure 3B, and the results were in agreement with the findings of TEM. The hydrodynamic size and distribution of PCSP-AuNPs (Figure 3C) were analyzed by the DLS method as 85.4 ± 2.2 nm with PDI of 0.276, indicating the relatively good dispersibility of PCAP-AuNPs in aqueous solution. The thickness of the capping layer contributes to the diversity in the size of the nanoparticles measured from the TEM and DLS analysis. This is because the size by the DLS method was analyzed in aqueous suspension, influenced by the solvent layer (Markus et al., 2017). When the absolute zeta potential is more than 30 mV, the stable suspension could be achieved (Kaasalainen et al., 2012). The zeta potential of PCSP-AuNPs was −35.7 ± 0.2 mV, indicating the satisfactory stability of the PCSP-AuNPs (Figure 3D).

#### 3.1.3. Composition of PCSP-AuNPs

To further characterize the PCSP-AuNPs, the FT-IR spectra was used to detect the active biomolecules in the PCSP-AuNPs (Figure 3E). The peak observed at 3405.55 cm^−1^ was ascribed to the stretching vibration of the phenolic isohydroxyl group. The C-H stretching vibration contributed to the absorption peak at 2927.88 cm^−1^ and the C = O stretching vibration might account for the peak at 1632.7 cm ^−1^. These two peaks, as well as the peak at 1729.47 cm^−1^ suggested that it was the endogenous reducibility of PCSP that enabled the reduction of HAuCl_4_ to PCSP-AuNPs (Hu et al., 2020; Zhang et al., 2021). The result for EDAX is displayed in Figure 3F, showing the presence of gold ions in the PCSP-AuNPs, which indeed validated the efficient formation of PCSP-AuNPs.

### 3.2. Immunomodulatory activities of PCSP-AuNPs in vitro

#### 3.2.1. No level

**Figure 4.**
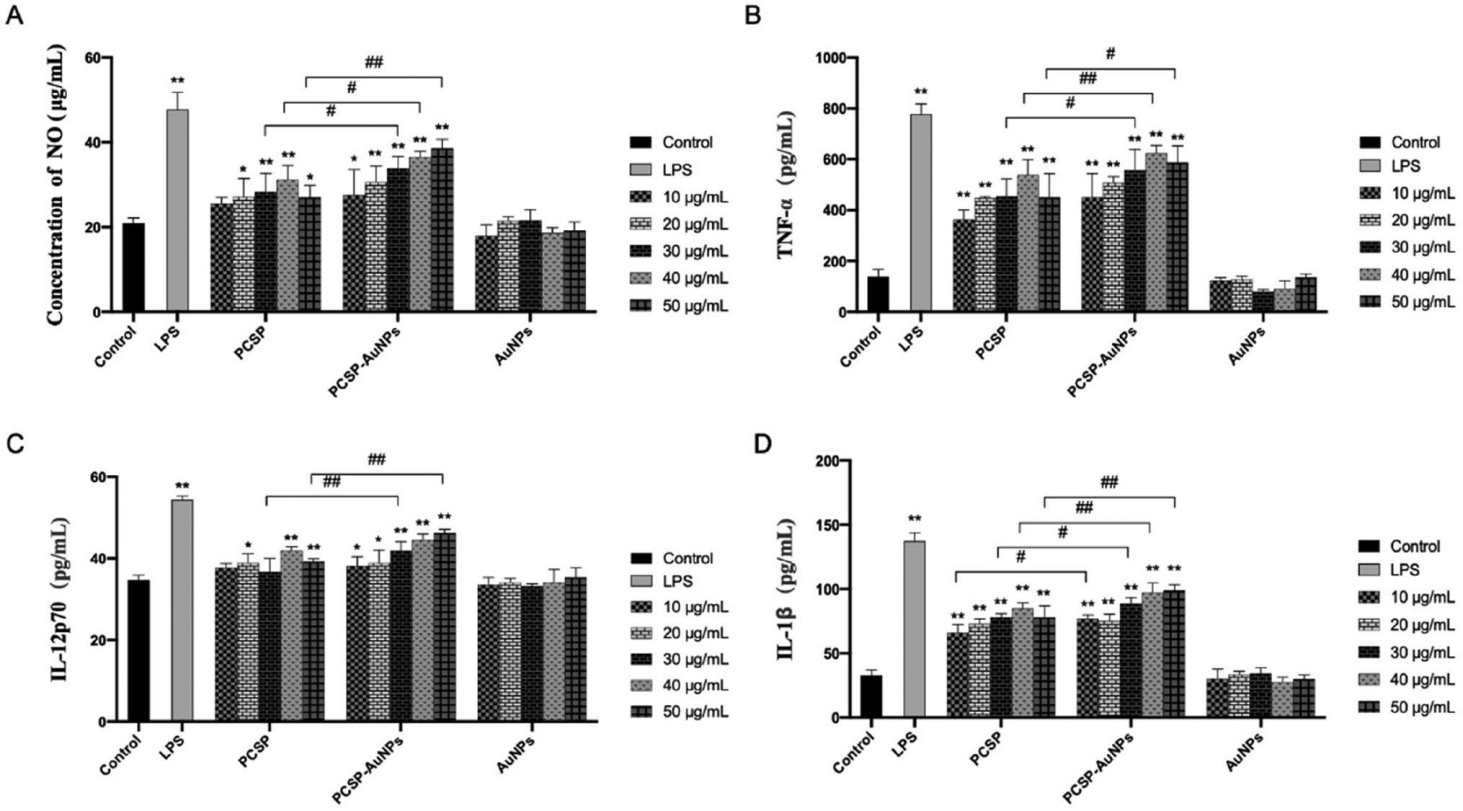
The activation of PCSP and PCSP-AuNPs to RAW264.7 *in vitro*. The secretion of NO (A), TNF-α (B), IL-12p70 (C) and IL-1β (D). *p < 0.05, **p < 0.01, compared to the control group; ^#^*p* < 0.05, ^##^*p* < 0.01, compared to the PCSP group.

In the immune system, nitric oxide (NO) has always been considered as the host’s first line of defense against infection, regulating the unfolding of a series of immune responses (García-Ortiz & Serrador, 2018). Therefore, we evaluated the intensity of NO production from macrophages to assess the *in vitro* immunomodulatory activity of PCSP-AuNPs. As illustrated in Figure 4A, PCSP significantly stimulate the release of NO in the range of 20 − 50 μg/mL concentration as compared to the blank control group, and reached the highest value at 40 μg/mL. The production of NO by PCSP-AuNPs increased with the growth of the concentration in the range of 10-50 μg/mL, and there was a remarkable increase at the higher concentration range (30 − 50 μg/mL), as compared to PCSP alone. The AuNPs without PCSP could not stimulate the secretion of NO. However, the NO level in the PCSP and PCSP-AuNPs group did not increase as much as in the LPS group. These results indicated that PCSP and PCSP-AuNPs effectively activated the macrophages *in vitro* without triggering inflammation.

#### 3.2.2. Secretion of cytokines

To further explore the effects of PCSP and PCSP-AuNPs on the immune response, we carried out the ELISA to detect the changes in the levels of cytokines in RAW264.7 macrophages. TNF-α is chiefly produced by tissue macrophages, inducing a series of innate immune responses during pathogenic invasion (Abdel-Maksoud et al., 2018). Interleukin-12 (IL-12), a heterodimeric cytokine produced by the APCs regulates lymphocyte activation and differentiation, and has long been studied for cancer immunotherapy (IL-12p70 belongs to the IL-12 family) (Nguyen et al., 2020; Glassman et al., 2021). And interleukin-1 beta (IL-1β) is a key cytokine in innate immunity, which recruits immune cells and regulates adaptive immune responses (Dinarello, 2009; Aarreberg et al., 2019). Therefore, we selected the above three cytokines to further study the role of PCSP-AuNPs in immunity. In Figure 4B–D, PCSP at concentrations of 10 and 30 μg/mL did not cause any obvious enhancement in the secretion of IL-12p70, while the other concentrations of PCSP and PCSP-AuNPs significantly stimulated the production of TNF-α, IL-12p70, and IL-1β in comparison to the control group (*p* < 0.05). However, none of them had as strong an effect as LPS did. Likewise, PCSP-AuNPs contributed more to the increase in the secretion of cytokines than PCSP mainly at higher concentrations and AuNPs alone had no effect on the production of the above cytokines.

### 3.3. Effect of PCSP-AuNPs on the viability of RAW264.7, L02 cells and HCC cell lines

**Figure 5.**
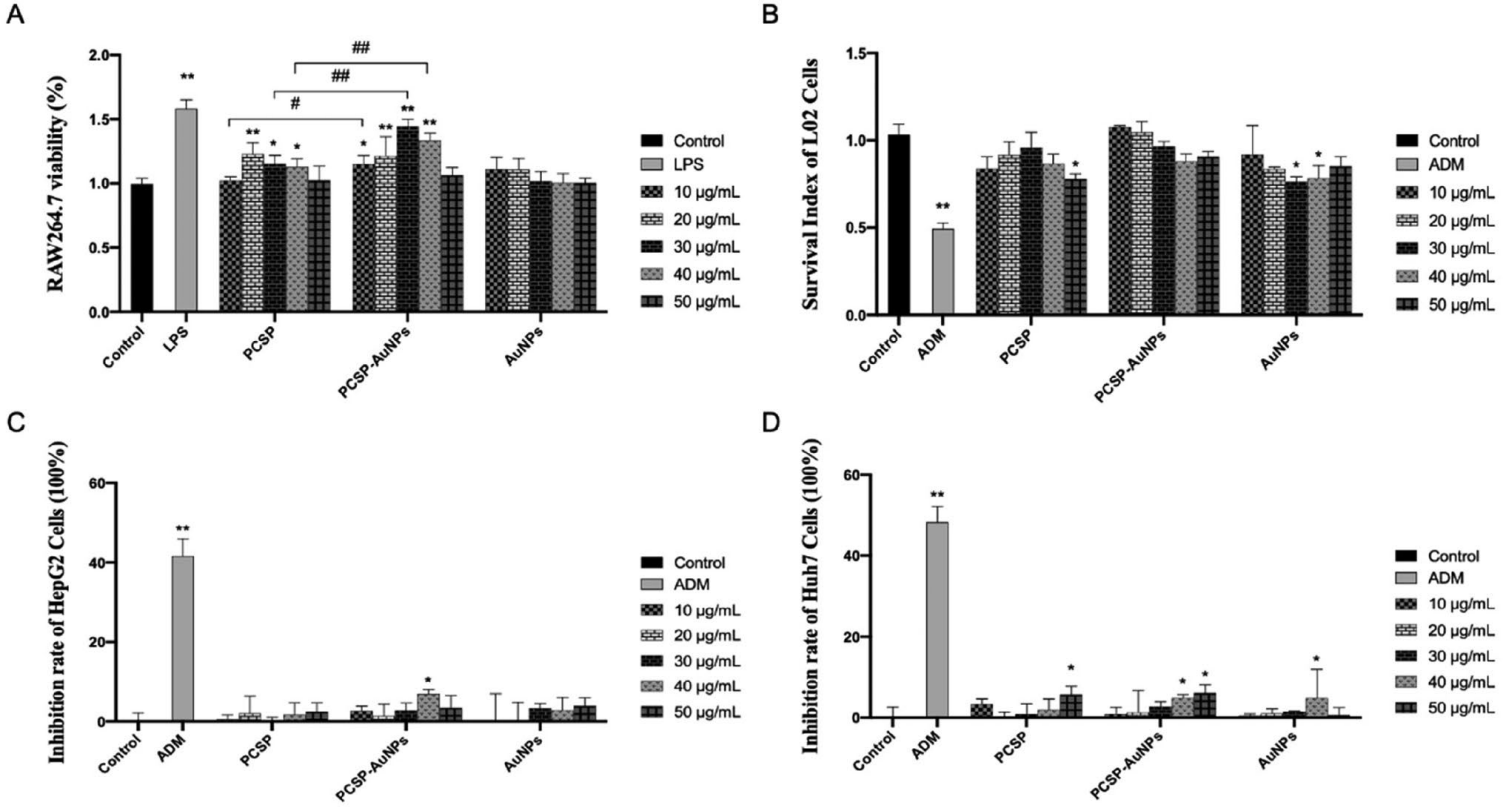
CCK-8 assay for detecting the effects of PCSP and PCSP-AuNPs on the viability of RAW264.7 (A), L02 cells (B), HepG2 cells (C), and Huh7 cells (D). *p < 0.05, **p < 0.01, compared to the control group; ^#^*p* < 0.05, ^##^*p* < 0.01, compared to the PCSP group.

Macrophages are the most plastic cells in the hematopoietic system and are present in all tissues with great functional diversity. They play important roles in development, homeostasis, tissue repair, and especially in immunity. They engulf pathogens to initiate innate immunity, and at the same time, they phagocytose products from the tumor microenvironment products to mediate anti-tumor immunity (Wynn et al., 2013; Chen et al., 2021). The viability of macrophages reflects the cytotoxicity of the drugs and its function is also related to the number of viable cells (Boechat et al., 2001). We used the CCK-8 test to assess the viability of RAW264.7 (Figure 5A). Both PCSP and PCSP-AuNPs stimulated the growth of RAW264.7 and PCSP-AuNPs was significantly stronger than PCSP at concentrations of 10, 30, and 40 μg/mL (*p* < 0.05). L02 cells are normal liver cells. Figure 5B demonstrated no apparent cytotoxic effect of PCSP and PCSP-AuNPs on L02 cells, except for the dose of PCSP up to 50 μg/mL. In conclusion, PCSP and PCSP-AuNPs showed good biocompatibility. Meanwhile, the effect of PCSP and PCSP-AuNPs on the growth of HCCs (HepG2 and Huh7 cells) was also detected by the CCK-8 kit. We found that PCSP and PCSP-AuNPs only had a certain inhibitory effect on HCCs at some high concentrations, but basically had no obvious influence under most conditions (Figure 5C and D). By the way, while ADM could effectively kill HCCs, it damaged nearly half of the L02 cells.

### 3.4. Antitumor efficacy of PCSP-AuNPs in vivo

#### 3.4.1. Body weight and tumor volume measurements

**Figure 6.**
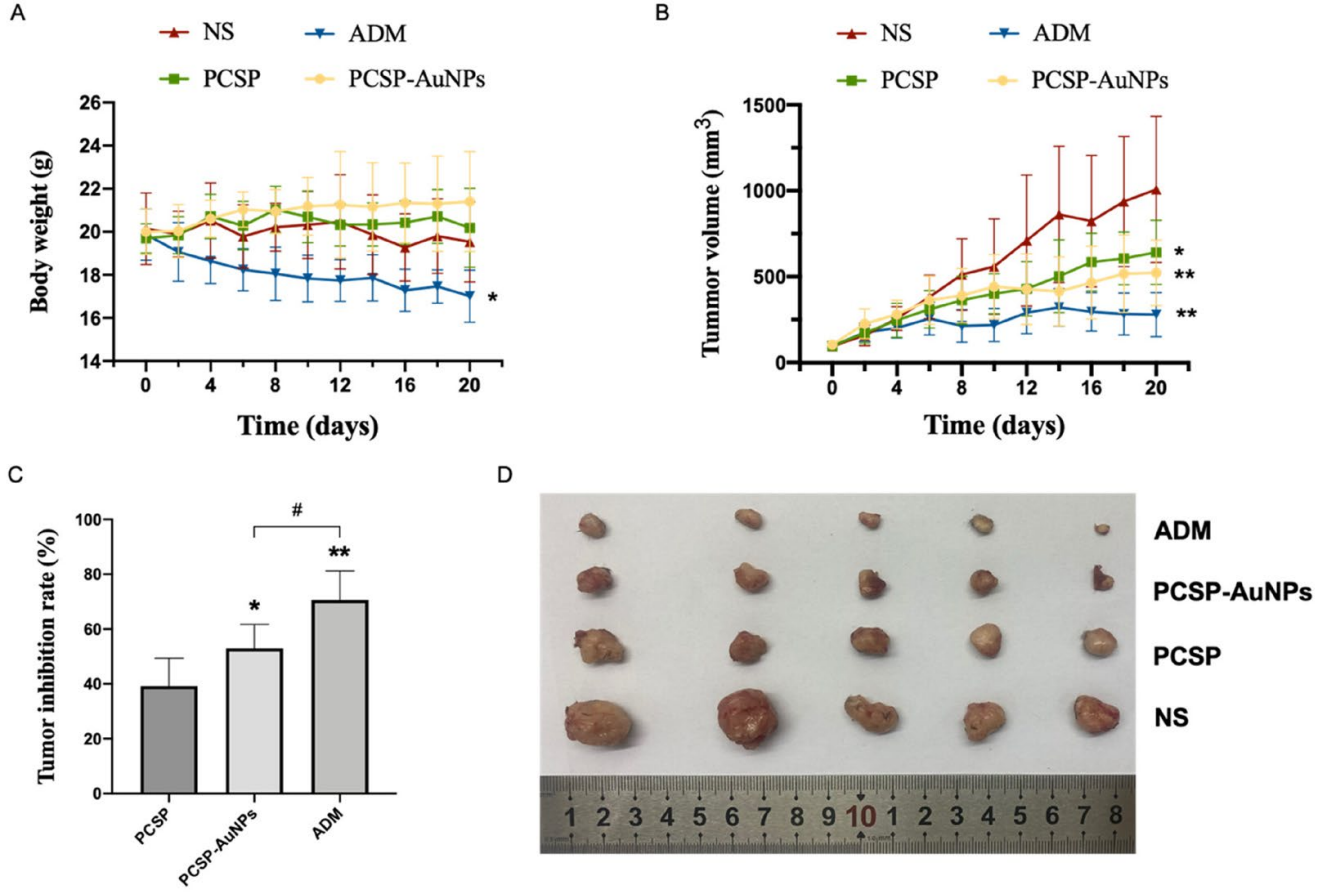
Antitumor effects of PCSP and PCSP-AuNPs on H22 tumor-bearing mice. (A) Body weights of mice as a function of time. (B) Tumor volume of mice during the 20 days. *p < 0.05, **p < 0.01, compared to the control group; ^#^*p* < 0.05, compared to the PCSP group. (C) Tumor inhibition rate in different treatments. *p < 0.05, **p < 0.01, compared to the PCSP group; ^#^*p* < 0.05, compared to the PCSP-AuNPs group. (D) Photograph of tumors from different groups.

In this experiment, the antitumor activity of PCSP and PCSP-AuNPs was determined using the traditional model, H22 tumor-bearing mice. The grouping and processing methods were as described before in the methods section. The body weight fluctuations in the mice were measured to assess their health status. Figure 6A depicted that the body weights of the mice treated with PCSP and PCSP-AuNPs were unaffected and even slightly increased compared to the NS group, implying that there was no noticeable damage caused by PCSP and PCSP-AuNPs. Figure 6B portrayed the trend for change in the tumor size. After the mice were sacrificed, the tumors were weighed to calculate tumor growth inhibition rates in each group (Figure 6C). To our excitement, the tumor growth in the PCSP-AuNPs group was remarkably suppressed and the tumor weight was also significantly reduced more than that of the NS group (*p* < 0.05). This may be due to the targeting ability of gold nanoparticles, which are an ideal delivery system for different drugs (Al-Dulimi et al., 2020), thus improving the bioavailability of PCSP-AuNPs. PCSP also displayed an apparent antitumor effect, but it was not as strong as the PCSP-AuNPs. Next, we took images of the tumors (Figure 6D), which were consistent with the previous results. Although ADM treatment strongly suppressed the tumor growth, it caused a dramatic weight loss and the mental state of the mice with ADM was significantly worse than in the other three groups, meaning the severe toxic side-effects of ADM on the mice.

#### 3.4.2. Immunohistochemical analysis of tumors treated with PCSP-AuNPs

**Figure 7.**
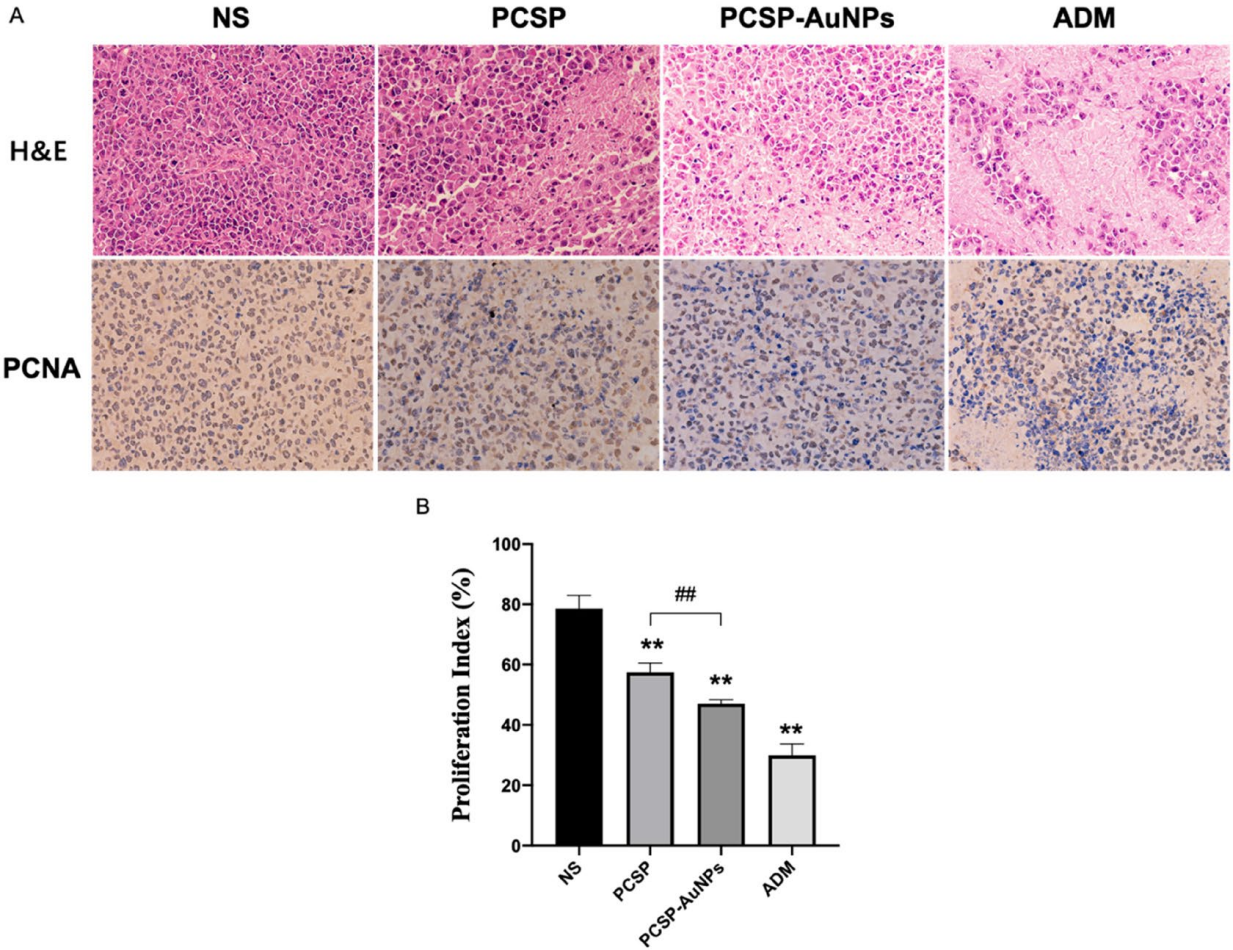
Pathological and histological observation of H22 tumors in mice. (A) H&E and PCNA staining of the tumors in different groups. (B) Proliferation index of different groups. *p < 0.05, **p < 0.01, compared to the control group; ^##^*p* < 0.01, compared to the PCSP group.

Subsequently, we carried out the H&E and PCNA staining of tumors to detect the anti-tumor efficacy of PCSP-AuNPs. Figure 7A revealed that PCSP-AuNPs generated more nuclear pyknosis and necrosis in the H&E staining. Furthermore, the PCNA-positive cells were considerably decreased in the PCSP-AuNPs group as compared to the NS group, which meant a decline in tumor proliferation. The positive expression of PCNA in the PCSP group also showed a sharp weakening, but not to the same extent as the PCSP-AuNPs group (Figure 7B). These results confirmed that the anti-cancer effects of PCSP-AuNPs were more potent than PCSP, which was consistent with the previous findings.

#### 3.4.3. Immunomodulatory effects of PCSP-AuNPs

**Figure 8.**
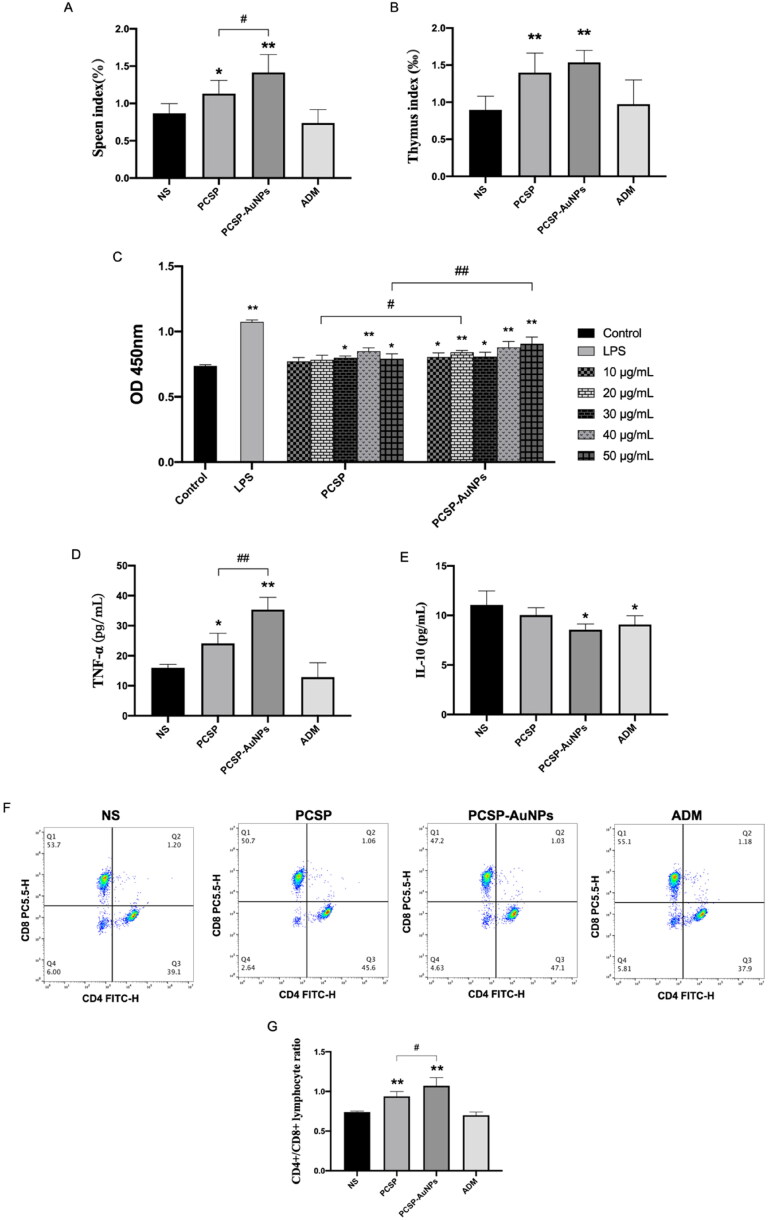
*In vivo* immune regulation mediated by PCSP and PCSP-AuNPs. (A, B) Spleen and thymus index in different groups. (C) Splenocyte proliferation treated with PCSP and PCSP at different concentrations. (D, E) Change in the level of cytokines (TNF-α and IL-10) in mice with different treatments. (F) The flow cytometry results of the CD4^+^/CD8^+^ ratio in peripheral blood. (G) The CD4^+^/CD8^+^ ratio in peripheral blood for each group. *p < 0.05, **p < 0.01, compared to the control group; ^#^*p* < 0.05, ^##^*p* < 0.01, compared to the PCSP group.

The immune system is an important mechanism in defending the host against pathogenic infection, and consists of the different immune organs, immune cells and immune molecules. Immune organs, such as the spleen and thymus, the main sites of the immune response, carry out protective responses to ensure that the noxious stimuli are eliminated (Blackburn & Kellems, 2005; Li et al., 2017; Xu et al., 2020). In the current study, we found that PCSP and PCSP-AuNPs could notably enlarge the spleen and thymus index in comparison to the NS group, and the PCSP-AuNPs performed better (Figure 8A and B) (*p* < 0.05). Lymphocytes proliferate in response to the stimulation of the immune system and play an important role in the immune response (Kuang et al., 2016). As displayed in Figure 8C, PCSP and PCSP-AuNPs showed the ability to promote splenic lymphocyte proliferation and when the concentrations were 20 and 50 μg/mL, PCSP-AuNPs exhibited a prominently higher efficacy than PCSP.

Next, we measured the levels of cytokines TNF-α and IL-10 in mice. Figure 8D revealed that PCSP-AuNPs markedly elevated the level of TNF-α relative to PCSP (*p* < 0.05), which was the same as *in vitro*. Nevertheless, it obviously downgraded the level of IL-10 (Figure 8E) (*p* < 0.05). IL-10 is predominantly secreted by the Th2 cells, which mainly works against the leukocytes, possessing major immunosuppressive functions (Wang et al., 2019). IL-10 is also known to limit the efficacy of cancer immunotherapy (Sawant et al., 2019). Therefore, it was a great thing that PCSP-AuNPs suppressed the level of IL-10.

In order to further understand the effect of PCSP-AuNPs on the improvement in immune function, we examined the ratio of CD4^+^/CD8^+^ T cells. It has been reported that a change in the ratio of CD4^+^/CD8^+^ lymphocytes was an indispensable immunological event after immune activation, and the increase in the percentage of CD4^+^/CD8^+^ T cells generally occurred in individuals with enhanced immunity (Lee et al., 2014; Wusiman et al., 2019). As expected, a significantly higher ratio of CD4^+^/CD8^+^ T cells was observed in the peripheral blood of mice injected with PCSP and PCSP-AuNPs than those treated with NS (Figure 8F and G). On the other hand, the above findings revealed the greater immunomodulatory capacity of PCSP-AuNPs than that of PCSP. The ADM group, however, did not induce any improvement in the ratio of CD4^+^/CD8^+^ T cells, but rather slightly reduced it. Taken together, combined with the results *in vitro* that PCSP and PCSP-AuNPs could hardly directly inhibit HCCs, we speculated that PCSP and PCSP-AuNPs may fight cancer by regulating the immune function.

#### 3.4.4. Biosafety of PCSP and PCSP-AuNPs

**Figure 9.**
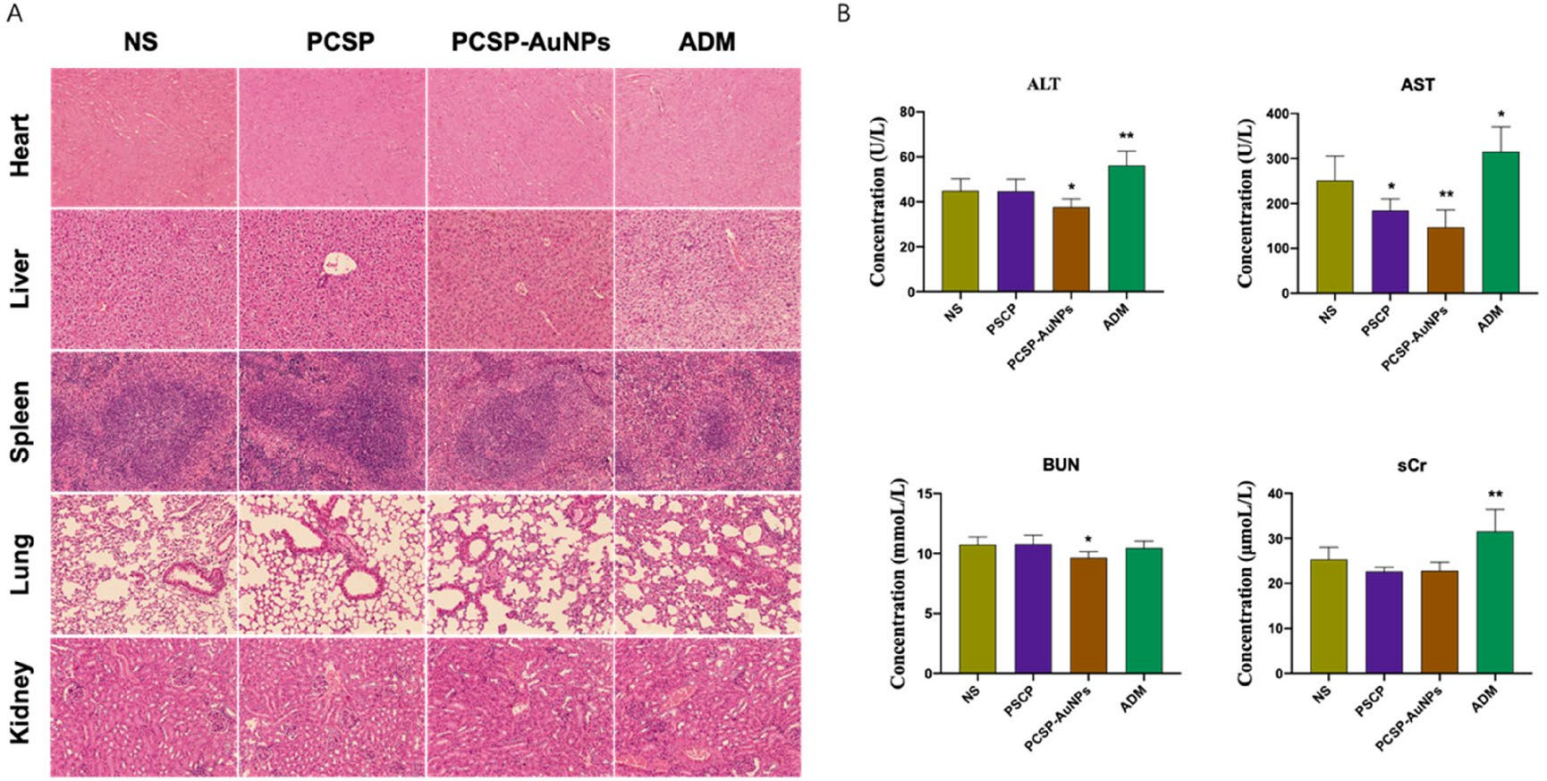
Toxicity tests. (A) H&E staining of the heart, liver, spleen, lung, and kidney. (B) The blood biochemical analysis for ALT, AST, Bun and sCr. *p < 0.05, **p < 0.01, compared to the control group.

Following the *in vitro* viability test with CCK-8 assay, we observed the vital organs of the mice by H&E staining and conducted liver and kidney function tests (ALT, AST, BUN, and sCr) to further verify the biological safety of PCSP and PCSP-AuNPs. H&E staining (Figure 9A) of the heart, liver, spleen, lung, and kidney showed that there were no obvious injuries caused by PCSP and PCSP-AuNPs. Biochemical results (Figure 9B) also expressed that PCSP and PCSP-AUNPs could alleviate the pressure on the liver and kidney. Nevertheless, both the H&E staining and biochemical analysis indicated that ADM had toxic side effects and caused distinct damage to the vital organs in the mice. In a word, our study opened a new possibility for the application of green synthetic nanomaterials for the safe and effective treatment of liver cancer.

## 3. Conclusion

In summary, the eco-friendly gold nanoparticles based on the extract from the TCM *Pseudobulbus Cremastrae seu Pleiones* were successfully produced and demonstrated to have efficacy against liver cancer with immunomodulatory functions *in vitro* and *in vivo*, which was stronger than PCSP alone. The physicochemical properties of PCSP-AuNPs were performed using UV-vis spectroscopy, TEM, AFM, DLS, FT-IR, and EDAX. The *in vitro* data revealed that PCSP-AuNPs could significantly stimulate the macrophages and induce the secretion of NO and cytokines. However, they could hardly directly suppress the proliferation of HCCs in the culture medium. In the H22 tumor-bearing mice, PCSP-AuNPs could suppress tumor growth, which was confirmed by the H&E and PCNA staining. And we suspected that the anti-cancer effects of PCSP-AuNPs were mediated through its immune-modulatory function since it caused an increase in CD4^+^/CD8^+^ ratio, spleen index, thymus index, and splenic lymphocyte viability, and altered the levels of immune-related cytokines in the mice. Thus, in conclusion, our findings have revealed a novel biocompatible nanocomposite for cancer immunotherapy.

## Ethics statement

All animal experiments and procedures followed guidelines approved by the Animal Care and Use Committee of Chongqing Medical University Institutions.
